# Recovery of Abdominal Muscle Thickness and Contractile Function in Women after Childbirth

**DOI:** 10.3390/ijerph18042130

**Published:** 2021-02-22

**Authors:** Mako Fukano, Yuka Tsukahara, Seira Takei, Sayaka Nose-Ogura, Tomoyuki Fujii, Suguru Torii

**Affiliations:** 1 College of Engineering, Shibaura Institute of Technology, Saitama 135-8548, Japan; 2 Faculty of Sport Sciences, Waseda University, Saitama 169-8050, Japan; shunto@waseda.jp; 3 Institute for Integrated Sports Medicine, Keio University School of Medicine, Tokyo 108-8345, Japan; yuka.voila@gmail.com; 4 Waseda Institute for Sport Sciences, Waseda University, Saitama 169-8050, Japan; 5 Orthopaedic Surgery, Sensory and Motor System Medicine, Graduate School of Medicine, The University of Tokyo, Tokyo 158-8557, Japan; saranaga0330@hotmail.com; 6 Waseda Institute of Human Growth and Development, Waseda University, Saitama 169-8050, Japan; 7 Department of Obstetrics and Gynecology, The University of Tokyo, Tokyo 158-8557, Japan; o-sayaka@hotmail.co.jp (S.N.-O.); fujiit-tky@umin.ac.jp (T.F.)

**Keywords:** postpartum period, abdominal muscles, ultrasound imaging, muscle thickness, thickness ratio

## Abstract

Abdominal muscles may be both morphologically and functionally affected by pregnancy. Dysfunction of the muscles can lead to persistent postpartum low back pain. The recovery process of the abdominal muscles following childbirth is not well understood. This study aimed to demonstrate the changes in the thickness and contractile function of abdominal muscles during the first six months postpartum. Nine perinatal and 15 nulliparous females participated. The thicknesses and contraction/relaxation thickness ratios of the rectus abdominis (RA), external abdominal oblique (EO), internal abdominal oblique (IO), and transverse abdominis (TrA) were measured using ultrasound images from 36–39 weeks’ gestation until six months postpartum. The RA, IO, and TrA muscles were thinner in perinatal females than controls at 36–39 weeks of gestation (4.8 vs. 9.47 mm (RA), 5.45 vs. 7.73 mm (IO), 2.56 vs. 3.38 mm (TrA), respectively). The thinner IO muscle persisted for six months after delivery. The decreased TrA thickness ratio persisted until four months post-delivery. Abdominal muscle thickness and contractile function decreased in the postpartum period. Therefore, abdominal muscle exercises might help prevent postpartum symptoms; however, because deterioration of muscle function is significant in the first four months, careful attention should be paid to exercise intensity. The study limitation was a relatively small sample size, thus future studies should involve more participants.

## 1. Introduction

Low back pain is a common complaint in women during pregnancy and the postpartum period, with approximately 20% of women reporting persistent pain for years [1]. Persistent low back pain may result in difficulty returning to daily life and sports activities after childbirth.

Muscular dysfunction of the abdominal muscles including the rectus abdominis (RA), external oblique (EO), internal oblique (IO), and transverse abdominis (TrA) is considered a contributing factor to low back pain during pregnancy and the postpartum period, because these muscles are critical in stabilising the spine [2]. Previous studies have reported that postpartum women with low back pain had lower endurance of the back flexor and extensor muscles and hip abduction muscles and lower muscle strength of the hip extensor muscles [3,4]. Furthermore, impairments in trunk flexor strength, muscle endurance, and steadiness of contraction for up to six months were reported in uncomplicated postpartum women [5]. These studies indicate the deficits in abdominal muscle function in postpartum women; however, the mechanisms responsible for impaired abdominal muscle function, such as the contractile function of these muscles contributing to muscle work, are unclear. Previous morphological studies reported that muscle length and thickness in abdominal muscles changed during pregnancy and the postpartum period; the length of the RA had increased by 115% of the normal length at 38 weeks of gestation to accommodate the growing foetus [6], and the RA [7,8] and IO muscles [8] were thinner immediately after childbirth in postpartum females than in nulliparous females. These data help understand the physiologic changes in pregnant and postpartum women; however, it is unknown when, after delivery, recovery from these changes occurs.

Postpartum health has been under-addressed by researchers, clinicians, and women themselves. although approximately 90% of postpartum women report at least one health problem [9]. In addition, the article stated that clinicians must initiate the discussion about postpartum health antenatally and encourage women to enlist needed support early in the postpartum period. Therefore, it is important to understand the changes of physical function from pregnancy to postpartum period to decrease morbidity and improve postpartum health.

Insights regarding the recovery process of physical function in postpartum women may contribute to the development of evidence-based postpartum exercise programs that can restore the pre-pregnancy state of the body and an active fitness level. The American College of Obstetricians and Gynecologists recommends aerobic and strength conditioning exercises before, during, and after pregnancy for women with uncomplicated pregnancies [10]. Physiologic changes during and after pregnancy should be considered when developing and/or prescribing exercise, especially regarding the intensity and amount settings; however, changes in the postpartum period are unknown although exercises are recommended. Therefore, the purpose of this study was to determine the changes in thickness and contractile function of abdominal muscles during pregnancy and the first six months after delivery and analyse the relationship between muscle thickness and muscle thickness ratio and maternal and foetal properties. This could provide insights into the recovery process of abdominal muscle functions in postpartum women and aid the development of evidence-based postpartum exercise programs. We hypothesized that abdominal muscle thickness and its function in postpartum women temporarily decrease, but gradually recover overtime.

## 2. Materials and Methods

### 2.1. Participants

This study was approved by the local institutional ethics committee and conducted in accordance with the Declaration of Helsinki. The purpose, procedures and risks of the study were presented to the participants, and written informed consent was obtained from each participant before beginning the study. A total of 9 perinatal females (PN group) and 15 nulliparous female controls (CTL group) participated in this study. The PN group participants had vaginal births at 38–40 weeks of pregnancy, were free from back and extremity pain in the 6 months before becoming pregnant, and had no history of serious injuries or any operative treatments. Participants were excluded if they had undergone an abdominal surgery, including a caesarean section. The CTL participants were nulliparous women of childbearing age and were free from back and lower extremity pain, a history of serious injuries or any operative treatments, and any subjective symptoms interfering with sports activities. The participants of both groups were involved various physical and/or recreational sports activities once or twice per week. There were no statistically significant differences in the age, height, and weight (pre-pregnancy weight for the PN group) between the two groups; the group demographics of the two groups are shown in Table 1. For the PN group, measurements were conducted at 36–39 weeks of gestation, 4–7 weeks after childbirth, and then every 4 weeks until 6 months postpartum.

### 2.2. Equipment

An ultrasound scanner (Sonosite MicroMaxx; Sonosite Inc., Bothell, WA, USA) with a 5 MHz linear probe was used. Ultrasound images of the RA, EO, IO, and TrA were obtained in B-mode, which produced two-dimensional cross-sectional greyscale images of these muscle groups.

### 2.3. Procedure

Ultrasound images were obtained with the participants in the supine position, with their hip and knees extended. The muscle thicknesses of the RA, EO, IO, and TrA muscles were obtained in the resting and draw-in conditions; the PN group was evaluated in the draw-in condition only after delivery. The draw-in is an isometric abdominal contraction and is commonly used as a clinical test to assess trunk muscle function in association with low back pain [11]. The participants were instructed on how to perform draw-in contractions and were asked to draw in their abdominal wall when in a motionless supine position. The probe was placed in the superior-inferior direction along the RA muscle fibres, and sagittal images were taken at three muscle bellies (L1, L2, L3) from the first superior RA belly. The muscle length of L1, L2, and L3 and the tendinous intersection length between L1 and L2 and L2 and L3 were measured in both conditions. The probe was placed transversely across the EO, IO, and TrA muscles at the umbilical level on their anterior axillary line. Data from the left side of the participants were analysed in this study. Three investigators, M. F., Y. T., and Se. Ta., captured the images and measured the muscle thicknesses. The reliability of agreement between the three investigators was assessed by calculating Fleiss’ Kappa from randomly selected images; κ = 0.714 was considered a substantial match.

The contractile functions were evaluated using the muscle thickness ratio, developed by Teyhen et al. [12], estimated from the contracted and relaxed muscle status of each muscle (muscle thickness ratio = thickness during contraction (draw-in)/thickness during relaxation). Changes in muscle thickness measured with ultrasound scanning is a valid index of muscle contraction [11], as compared with electromyography [13] or magnetic resonance imaging [14].

### 2.4. Statistical Analyses

All statistical analyses were performed using statistical software (SPSS version 25; IBM Corp., Armonk, NY, USA). Differences between the groups (PN and CTL) were determined for participants’ characteristics using the Mann–Whitney U test. The Kruskal–Wallis test was performed to determine whether significant differences existed in terms of muscle thickness, muscle length of RA, tendinous intersection length of RA, and muscle thickness ratio between the PN and CTL groups, at each time point. If statistical significance was determined, a post hoc test (Dunn–Bonferroni test) was performed. Spearman’s rank correlation coefficients were used to assess correlations between age, body mass index, number of births, foetal weight at birth, and the muscle thicknesses and muscle thickness ratios at 6 months after childbirth. Data are described as mean ± standard deviations. Statistical significance was set at *p* < 0.05.

## 3. Results

Table 2 compares the RA, EO, IO, and TrA muscle thicknesses of the PN group with those of the CTL group. Kruskal–Wallis test indicated statistical significance for RA (H = 29.016; *p* < 0.001), IO (H = 21.360; *p* = 0.003), and TrA (H = 21.281; *p* = 0.003) thicknesses; the RA, IO, and TrA muscles at 36–39 weeks of gestation in the PN group were thinner than that in the CTL group, and the thinner IO muscle persisted for six months after delivery. The muscle length of L1 (H = 19.119; *p* = 0.008) and tendinous intersection length between L2 and L3 at 36–39 weeks of gestation in the PN group (H = 18.902; *p* = 0.009) were significantly longer than those in the CTL group.

Table 3 shows the comparison of the muscle thickness ratio between the two groups. Kruskal–Wallis test indicated a statistically significant difference in the thickness ratio of the TrA muscle from 1 month post-delivery and for four months post-delivery (H = 17.409; *p* = 0.008).

Table 4 presents the Spearman’s rank correlation coefficients between the muscle thicknesses and muscle thickness ratios at six months after childbirth and maternal and foetal factors. No significant correlations were identified.

## 4. Discussion

The present study shows muscle thickness recovery and change in contractile functions of abdominal muscles without any intervention after vaginal delivery; the thickness of the RA muscle increased within 1 month, while the thinner IO muscle needed more than six months to recover after delivery. The contractile function of the TrA muscle decreased for at least four months after delivery. To the best of our knowledge, this is the first longitudinal study to examine changes in the thickness and contractile functions of abdominal muscles during the first six months after vaginal birth.

Significant stretching was observed in RA (L1) and tendinous intersection length between L2 and L3 at 36–39 weeks of gestation in the PN group; the L2 and L3 and L1–L2 lengths were likely to be stretched but the changes were not statistically significant. This result indicated that the RA stretch was accompanied by increased abdominal circumference due to foetal growth and stretch-induced changes in abdominal muscles during pregnancy. The skeletal muscles can adapt to chronic length change by a change in the number of serial sarcomeres [15,16]. Some studies reported increased sarcomere lengths, rather than sarcomere numbers, that occur as an incomplete or partially unsuccessful adaptation to chronic stretching [17,18]. In either case, muscle functional properties may change after morphological adaptation to chronic stretching. A previous animal study demonstrated that the serial sarcomere number increased after applied chronic muscle stretching by tendon transfer but muscle excursion decreased in the extensor digitorum muscle of the second toe [19], although the serial sarcomere number is proportional to muscle excursion under normal conditions [20]. It is hypothesised that chronic and incremental mechanical stretching may induce morphological adaptation (i.e., the number or length of sarcomeres increases, and functional properties related to muscle contraction might be altered in the RA of the PN group). Further studies addressing muscle functions after morphological adaptation during pregnancy are needed.

**Figure 1:**
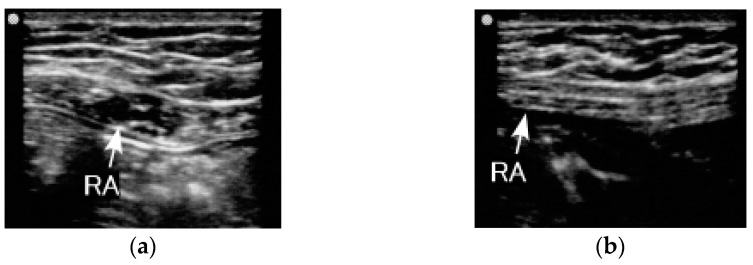
Rectus abdominus (RA) of a perinatal woman. The “wave-like” shape can be observed in the (**a**) image. (**a**) This image was taken one month after delivery; (**b**) this image was taken four months after delivery.

The thicknesses of the RA and TrA muscles in the PN group spontaneously returned to pre-pregnancy state one month after childbirth and were comparable with the thicknesses of the same muscles in the CTL group. Recovery of the thinner IO muscle in the PN group was consistent with findings of a previous study [8]. The RA muscle thickness after delivery, which was approximately 8 mm, was also consistent with previous reports, within the first month of delivery [8]; on the first day and at eight weeks, six months, and 12 months [7]; and at eight weeks and 26 weeks [5] after delivery. These findings indicate that the RA thickness promptly returned to approximately 8 mm after delivery. On the other hand, a “wave-like” shape was observed on the ultrasound images on the longitudinal axis of the RA muscle during one to three months after childbirth (Figure 1). This finding suggests that a decrease in RA muscle tension occurred by delivery of the foetus, amnion fluid, and placenta since the RA was stretched and lengthened by pregnancy. A previous ultrasound study showed an obvious increase of width and cross-sectional area of the RA one day after childbirth.

The authors hypothesised that the changes occurred from stretch-induced selective hypertrophy of Type I fibres [7]; however, it could also be a mere reaction to a rapid decrease in mechanical tension following delivery of the foetus. The rapid decrease in RA tension can cause the muscle to be incapable of generating or transferring force due to redundant and/or shortened length of serial muscle sarcomeres from a resting length in a length-tension relationship. A previous study reported that the ability to perform a curl-up, which generally declines starting around the twenty-sixth week of pregnancy, increased to moderate success or success in five subjects, among six people, eight weeks after birth [6]. On a subcellular scale, previous studies have demonstrated that the serial sarcomere number reduced, and muscle shortening occurred in response to understretching [21,22]. During adaptation to understretching, degeneration of fibres (i.e., dissolution of myofibrils) progress over the first few weeks, and new myofibril formation and restoration of normal sarcomere lengths occur by four weeks [22]. Collectively, it can be considered that two to three months period after delivery is a critical morphological recovery period, during which the lengthened RA muscle is restored to its original length. However, the ultrasound image (i.e., “wave-like” shape) was not a quantitative finding; quantitative measurements of muscle adaptation and muscle force generation and transfer are required for further analysis. In addition, previous studies reported that muscle atrophy was induced by a change in muscle length [23,24], and muscle atrophy was more severe when the muscles were fixed in the shortened position, rather than in the stretched position [25,26]. Therefore, strength conditioning exercises especially on the RA after pregnancy should be emphasised more for women with uncomplicated pregnancies and deliveries.

In this study, the decreased contractile function of the TrA persisted for the first four months after delivery without any intervention. This suggests neuromuscular deterioration of the TrA; changes in thickness can indicate changes in the electrical activity in the muscle [27]. The TrA muscle thickness ratio of the CTL group was comparable or slightly higher than that in previous studies, approximately 1.5 [14,28,29], whereas the ratio of the PN group was significantly lower, around 1.1. In our results, thickness changes in TrA and IO were observed during draw-in; this is consistent with studies observing muscle activation [14,28,29]. Therefore, it is supposed that TrA and IO activate co-operatively. In this study, the IO may compensate for the underuse of TrA in the PN group; the percentage of IO thickness to the total muscle thickness of EO, IO, and TrA during contraction was higher in the PN group than in the CTL group (PN group (at four months): EO, 46.7%; IO, 32.3%; TrA, 21.0%; CTL group: EO, 46.8%; IO, 25.4%; TrA, 27.8%). A previous review article stated that compensatory overuse of EO instead of underuse of TrA is a common trunk control strategy in postpartum women [30]; however, compensatory overuse of the IO can be another strategy for trunk control. In addition, ultrasound measurement is unable to detect the activity of EO since its thickness does not change consistently with muscle contraction, whereas the thicknesses of IO and TrA correlated with EMG activities for low-level isometric contractions [13]. Therefore, further estimation by a method other than ultrasound will be needed to estimate compensation strategies for underuse of TrA in the lateral abdominal muscles.

The decreased TrA function could constitute a significant risk for low back pain. One magnetic resonance imaging study found that the TrA muscle improves the stabilisation of the lumbopelvic region by tightly forming musculofascial bands that accompany TrA contraction during the draw-in condition [14]. Lower TrA thickness ratio was reported in patients with low back pain than in those in the control group [28]. Recently, it has been found that the contractile function of the TrA muscle is also associated with postnatal urinary incontinence; women with postnatal urinary incontinence have a lower TrA thickness during muscle contraction than nulliparous women and those without urinary incontinence [31]. Therefore, exercising the lateral abdominal muscles might help prevent various symptoms in postpartum women and can improve their quality of life.

These findings also provide practicing clinicians and/or exercise instructors insights into physical conditions of postpartum women. The study suggests that careful attention should be paid to the activity level; improper or excessive high-intensity and/or heavy load training exercises should be avoided in the first four months postpartum because deterioration of abdominal muscle function has been noticed in this period. Additionally, all postpartum women need to be careful with strength and load-setting while exercising because no significant correlations were identified between muscle thickness and muscle thickness ratios at six months after childbirth and maternal and foetal factors. Further exercise investigation studies are necessary for developing appropriate exercise programs to recover and/or improve abdominal contractile functions, especially those of the TrA muscle, in postpartum women.

One limitation is that we used ultrasound images to assess the change in muscle thickness to measure the contractile function during the draw-in manoeuvre. Ultrasound images can detect muscle thickness changes and its forms but is unable to determine neuromuscular function, such as muscle activation. Besides, some women had forgotten how to contract their abdominal muscles during the draw-in contractions, immediately after to two to three months after childbirth. Further research is needed to assess the recovery of abdominal muscles from neuromuscular functions. Another limitation is the relatively small sample size. Future studies should involve more participants and should also investigate postpartum women who gave birth by caesarean sections because the rate of caesarean sections is increasing globally [32].

## 5. Conclusions

The present study found that the abdominal muscle thickness and contractile function decreased during pregnancy and the postpartum period and these changes were independent of maternal and foetal factors. These morphological changes and functional deterioration of the abdominal muscles from pregnancy to the postpartum period should be considered when postpartum women exercise. Future studies will facilitate the development of an optimal postpartum exercise program that prevents various symptoms in postpartum women and improves their quality of life while recovering abdominal muscle functions.

## Figures and Tables

**Table 1:** Demographic data of perinatal and control groups.

Group	PN		CTL		
	(*n* = 9)		(*n* = 15)		
Parameter	Mean	SD	Mean	SD	*p*-Value
Height (cm)	162.4	2.8	161.3	6.1	0.86
Weight (kg) ^1^	53.5	5.2	53.8	5.2	0.97
Age (years)	33.2	5.2	29.4	4.1	0.12

^1^ Weight before pregnancy for perinatal females; SD, standard deviation; PN, perinatal group; CTL, nulliparous female control group.

**Table 2:** Comparison of abdominal muscle thickness and length between perinatal and control groups.

Group	CTL		PN													
			36–39 weeks		1 month		2 months		3 months		4 months		5 months		6 months	
Muscle	Mean	SD	Mean	SD	Mean	SD	Mean	SD	Mean	SD	Mean	SD	Mean	SD	Mean	SD
Muscle thicknesses (mm)																
RA	9.47	1.44	4.80	0.84 *	8.49	2.10	8.20	1.84	7.87	1.35	7.41	1.14	7.20	1.06	8.00	1.32
EO	4.87	1.44	3.70	1.17	4.70	1.28	5.33	1.45	4.98	1.68	4.90	1.59	5.06	1.67	5.16	1.45
IO	7.73	1.71	5.45	1.58 *	5.58	0.94 *	6.06	1.03 *	6.56	1.88 *	6.20	1.35 *	7.13	2.35	6.36	1.60 *
TrA	3.38	1.10	2.56	0.54 *	4.32	2.94	3.14	0.69	4.18	2.64	3.40	0.59	3.27	0.53	2.99	0.39 *
Muscle length (mm)																
RA (L1)	57.7	22.7	94.5	20.6 *	63.3	11.7	59.7	12.5	53.4	11.3	56.9	12.0	54.7	51.4	10.7	10.7
RA (L2)	66.1	8.3	80.8	26.9	60.8	8.6	60.0	15.2	58.9	9.5	59.3	14.3	58.8	13.5	60.2	12.1
RA (L3)	64.5	8.3	73.5	12.0	58.3	10.4	53.8	4.8	57.7	13.1	47.7	4.6	52.5	9.6	59.2	12.6
Tendinous intersection length																
L1-L2	12.1	4.4	19.8	6.6	14.3	3.1	14.0	4.0	12.4	5.9	10.7	4.1	11.1	3.2	12.6	4.7
L2-L3	10.6	3.0	20.0	7.1 *	12.8	2.0	13.7	4.3	11.0	2.5	11.1	4.7	14.3	5.1	13.7	4.5

* Statistical significance (vs. Control group; *p* < 0.05), CTL, nulliparous female control group; PN, perinatal group; SD, standard deviation; RA, rectus abdominis; EO, external abdominal oblique; IO, internal abdominal oblique; TrA, transverse abdominal; L1, first superior belly of RA; L2, second superior belly of RA; L3, third superior belly of RA.

**Table 3:** Comparison of abdominal muscle thickness ratio between perinatal and control groups.

Group	CTL		PN											
			1 Month		2 Months		3 Months		4 Months		5 Months		6 Months	
Muscle	Mean	SD	Mean	SD	Mean	SD	Mean	SD	Mean	SD	Mean	SD	Mean	SD
RA	1.12	0.15	1.09	0.04	1.18	0.16	1.17	0.23	1.21	0.18	1.14	0.20	0.26	0.21
EO	1.11	0.28	1.18	0.76	0.96	0.37	1.02	0.39	1.12	0.35	1.03	0.38	1.12	0.39
IO	1.21	0.23	1.13	0.13	1.29	0.34	1.11	0.31	1.28	0.39	1.26	0.31	1.22	0.32
TrA	1.68	0.56	1.07	0.20 *	1.13	0.20 *	1.18	0.19 *	1.02	0.26 *	1.26	0.36	1.40	0.59

* Statistical significance (vs. Control group; *p* < 0.05); CTL, nulliparous female control group; PN, perinatal group; SD, standard deviation; RA, rectus abdominis; EO, external abdominal oblique; IO, internal abdominal oblique; TrA, transverse abdominal.

**Table 4:** Correlations between muscle thickness and muscle thickness ratios at six months after childbirth and maternal and foetal factors.

	Muscle Thickness								Muscle Thickness Ratio							
	RA		EO		IO		TrA		RA		EO		IO		TrA	
	*r*	*p*	*r*	*p*	*r*	*p*	*r*	*p*	*r*	*p*	*r*	*p*	*r*	*p*	*r*	*p*
Age	0.36	0.38	0.37	0.37	0.30	0.46	0.24	0.55	0.32	0.43	0.59	0.12	0.41	0.31	0.27	0.56
Body mass index	0.48	0.23	0.35	0.40	0.28	0.51	0.44	0.27	−0.12	0.78	0.21	0.61	0.50	0.21	0.04	0.94
Number of births	0.33	0.43	0.54	0.16	0.06	0.90	0.22	0.60	0.00	1.00	0.22	0.60	0.00	1.00	0.29	0.53
Foetal weight	−0.24	0.57	−0.32	0.43	0.16	0.71	0.14	0.73	0.42	0.29	0.71	0.07	0.50	0.21	0.57	0.18

*r*, Spearman’s rank correlation coefficient; *p*, probability (two-tailed significance); RA, rectus abdominis; EO, external abdominal oblique; IO, internal abdominal oblique; TrA, transverse abdominal.

## Data Availability

The data presented in this study are available on request from the corresponding author.
